# Influence of the COVID-19 Outbreak in Vulnerable Patients (Pediatric Patients, Pregnant Women, and Elderly Patients) on an Emergency Medical Service System: A Pre- and Post-COVID-19 Pandemic Comparative Study Using the Population-Based ORION Registry

**DOI:** 10.3390/medicina60020345

**Published:** 2024-02-19

**Authors:** Koshi Ota, Masahiko Nitta, Tomonobu Komeya, Tetsuya Matsuoka, Akira Takasu

**Affiliations:** 1 Department of Emergency and Critical Care Medicine, Osaka Medical and Pharmaceutical University, Takatsuki 569-8686, Japan; nittam@ompu.ac.jp (M.N.); akira.takasu@ompu.ac.jp (A.T.); 2 Working Group for Analysis of the Emergency Medical Care System in Osaka Prefecture, Osaka 530-0000, Japan; t-matsuoka@rgmc.izumisano.osaka.jp; 3 Osaka Prefectural Government, Osaka 540-8570, Japan; komeyat@mbox.pref.osaka.lg.jp

**Keywords:** COVID-19, child, elderly patient, difficult-to-transport case, pregnant woman

## Abstract

*Background and Objective:* Coronavirus disease 2019 (COVID-19), caused by severe acute respiratory syndrome coronavirus 2 (SARS-CoV-2), has spread all over the world. To assess the influence of the COVID-19 pandemic on emergency medical services (EMS) for vulnerable patients transported by ambulance. *Materials and Methods:* This study was a retrospective, descriptive study with a study period from 1 January 2019 to 31 December 2021 using the Osaka Emergency Information Research Intelligent Operation Network (ORION) system. We included all pediatric patients, pregnant women, and elderly patients ≥ 65 years of age transported by ambulance in Osaka Prefecture. The main outcome of this study was difficult-to-transport cases. We calculated the rate of difficult-to-transport cases under several conditions. *Results*: For the two year-long periods of 1 January 2019 to 31 December 2019 and 1 January 2021 to 31 December 2021, a total of 887,647 patients were transported to hospital by ambulance in Osaka Prefecture. The total number of vulnerable patients was 579,815 (304,882 in 2019 and 274,933 in 2021). Multivariate logistic regression analysis showed that difficult-to-transport cases were significantly more frequent in 2021 than in 2019. Difficult-to-transport cases were significantly less frequent in the vulnerable population than in the non-vulnerable population (adjusted odds ratio 0.81, 95% confidence interval 0.80–0.83; *p* < 0.001). *Conclusion*: During the pandemic (2021), difficult-to-transport cases were more frequent compared to before the pandemic (2019); however, vulnerable patients were not the cause of difficulties in obtaining hospital acceptance for transport.

## 1. Introduction

Coronavirus disease 2019 (COVID-19) is caused by severe acute respiratory syndrome coronavirus 2 (SARS-CoV-2) and was first identified in Wuhan, China, in December 2019. Since then, the COVID-19 outbreak has spread all over the world [1]. The World Health Organization (WHO) declared COVID-19 a pandemic on 11 March 2020.

A previous study showed the impact of the COVID-19 pandemic on emergency medical services (EMS) systems in Osaka City, Japan [2]. Compared to 2018 or 2019, the proportion of cases experiencing difficulty in obtaining hospital acceptance in Osaka Prefecture among patients transported by ambulance due to acute disease was increased in 2020 [3,4]. However, few reports about the proportion experiencing difficulty with hospital acceptance were available in 2021, and to the best of our knowledge, no studies on the impact of the COVID-19 pandemic on EMS systems for vulnerable patients including pediatric patients, pregnant patients, and elderly patients have been reported [5].

Difficulty in obtaining hospital acceptance for the transfer of vulnerable patients (difficult-to-transport cases) has been a problem in public healthcare systems, not only in Osaka Prefecture but throughout Japan [6]. Previous studies for pediatric patients and pregnant patients revealed that there were few barriers of the healthcare system for emergency transportation before and after the COVID-19 pandemic, [3,4]. However, there were difficult-to-transport cases for elderly patients because they had a lot of comorbidities. Vulnerable populations, particularly elderly people, have made up increasingly large proportions of the global population, and transportation by ambulance has been increasing [7,8]. In addition, elderly patients are more likely to develop COVID-19 and experience more critical or mortal conditions than the general population [9,10]. Previous studies have revealed that vulnerable patients other than the elderly were not associated with difficult-to-transport cases in 2020 compared with 2018 or 2019 [3,4]. As the number of COVID-19 patients was small in the first half of 2020, comparisons between before and during the COVID-19 pandemic need to be made using 2019 and 2021 data. Three waves (the third, fourth, and fifth waves) of the COVID-19 outbreak occurred in 2021, and difficult-to-transport cases became a problem and caused a medical crisis in Osaka Prefecture in 2021.

This study aimed to assess the influence of the COVID-19 pandemic on the EMS system by comparing the pre-pandemic year of 2019 and the post-pandemic year of 2021, with a focus on vulnerable patients transported by ambulance in Osaka Prefecture.

## 2. Methods

### 2.1. Study Design and Setting

This study was a retrospective, descriptive study with a study period from 1 January 2019 to 31 December 2019 and from 1 January 2021 to 31 December 2021 using the Osaka Emergency Information Research Intelligent Operation Network (ORION) system [11]. Osaka Prefecture, the largest metropolitan community in western Japan, has a population of about 8.8 million and a total area of 1905 km^2^. To assess the influence of the COVID-19 pandemic on the EMS system, we focused on vulnerable patients transported by ambulance in Osaka Prefecture. Pediatric patients were divided into four age groups: infants, 0 years; toddlers, 1–4 years; children, 5–9 years; and adolescents, 10–14 years [4]. We defined “women of childbearing age” as female patients aged 15–44 years [12]. We used the presumptive diagnosis and final diagnosis for patients who were admitted, using the International Classification of Diseases, 10th revision (ICD-10) [13]. The present study defined patients with pregnancy-related issues (pregnant patients) as ICD-10 codes O00–O99 and P00–P96. We also classified elderly patients according to the Japan Gerontological Society: pre-old, 65–74 years; old, 75–89 years; and super-old, ≥90 years [14]. We collected “COVID-19” data as ICD-10 code U07.1, and “COVID-19 suspected” data (if the presence of the virus was not confirmed) as U07.2.

This study excluded patients who were not transported to a hospital. We also excluded patients who were transported in 2020, because only 92 COVID-19 patients were encountered between January and June 2020, and we wanted to examine an entire year influenced by COVID-19. Ambulance records in Osaka Prefecture are considered administrative records, so the need to obtain informed consent from participants was waived because the data were anonymous. This study was approved by the Ethics Committee of Osaka Prefectural Government (Osaka City, Japan). Strengthening the Reporting of Observational studies in Epidemiology (STROBE) guidelines were used to design and report the results of this study, and the Reporting of Studies Conducted using Observational Routinely Collected Health Data (RECORD) statement was also used to design and report the results of this study [15,16].

### 2.2. The Emergency Medical Service (EMS) System in Japan

When emergency patients call for EMS at the scene, on-scene EMS personnel assess the patient’s condition and then transport the patient to a hospital that can accept and treat the patient [4]. Only after obtaining permission from the selected hospital via a phone call can ambulances transport the patient to the hospital [4]. All expenses are covered by local governments, and there is no charge to the patient for care and transportation [4]. Recently, the number of cases of emergency patients’ transportation to a hospital by EMS has been increasing and exceeding the hospital capacity [4]. Therefore, there has been increasing cases of refusal to provide emergency transportation.

### 2.3. The ORION System

The Osaka prefectural government developed and introduced the ORION system as an information system for emergency patients that uses a smartphone application for hospital selection by on-scene EMS personnel. The ORION system collects all ambulance records. Medical institutions obtain information on the diagnoses and outcomes of patients transported there, and the ORION system has merged these data with ambulance records, including smartphone application data, since January 2015.

### 2.4. Data Collection and Quality Control

Data were uniformly collected using specific data collection forms and included the reason for the ambulance call, location of the accident, time of day, day of the week, and tools used, in addition to age, sex, and ICD-10 code. The details of the situation, including the name of the fire department, the accident type, the date of ambulance dispatch, a detailed time course before hospital arrival (time of call, ambulance dispatch, arrival at the scene, patient communication, patient’s condition in the ambulance, time of departure from the scene, and time of hospital arrival), and patient information, were recorded in text form. These data were completed by EMS personnel, then transferred to the information center in the Osaka Municipal Fire Department. To ensure the quality of the data, data sheets showing incomplete information were re-submitted to the relevant EMS personnel for correction.

### 2.5. Availability of Data and Materials

The datasets used in the current study are available from the corresponding author on reasonable request.

### 2.6. Outcomes

The primary outcome of this study was difficulty in obtaining hospital acceptance for transfer of a patient. Cases where this occurred (difficult-to-transport cases) were defined, according to the guidelines of the Fire and Disaster Management Agency [17], as those cases in which the interval from arrival at the scene to departure from the scene was longer than 30 min or cases for which ambulance crews needed to make four or more phone calls to hospitals before obtaining hospital acceptance. Disposition at the emergency department (ED), such as emergency admission, discharge to home, transfer to another hospital, or death in the ED, was also collected.

### 2.7. Data Analysis

We calculated the numbers of patients transported by ambulance due to any cause for the year from 1 January to 31 December 2021. For the purposes of comparison, the numbers of patients transported by ambulance for the same reasons for the year from 1 January to 31 December 2019 were also collected. In addition, we used logistic regression analysis to calculate the rate of difficulty with hospital acceptance for patients in the two year-long periods studied, then calculated the crude odds ratio (OR) for each year for difficult-to-transport cases along with the 95% confidence interval (CI), with 2019 as the reference. The OR and 95%CI of difficult-to-transport cases according to month (as compared with June), sex, weekends (as compared with weekdays), nighttime (17:00–09:00) (as compared with daytime), and each category of vulnerability were estimated using multivariate analyses. These variables were chosen based on the previous studies [3,4].

All data were statistically analyzed using SPSS version 25.0 software (IBM Corp., Armonk, NY, USA) or STATA (version 16.1; Stata Corp., College Station, TX, USA). All tests were two-tailed, with values of *p* < 0.05 considered statistically significant.

## 3. Results

Baseline Characteristics

For the two year-long periods of 1 January 2019 to 31 December 2019 and 1 January 2021 to 31 December 2021, a total of 948,248 patients were transported to hospitals by ambulance in Osaka Prefecture, Japan. Of these, 887,647 were enrolled in the present study, after excluding 60,601 patients transferred to a different hospital. Each year, 468,699 patients in 2019 and 418,950 patients in 2021 were transported to hospitals by ambulance (Table 1). The total number of pediatric patients (0–14 years old) was 65,107 (7.3%; 37,547 (8.0%) in 2019 and 27,560 (6.6%) in 2021; *p* < 0.001). The total number of women of childbearing age (15–44 years old) was 79,335 (8.9%; 43,105 (9.2%) in 2019 and 36,230 (8.6%) in 2021; *p* < 0.001). In addition, the total number of pregnant women was 1644 (0.2%; 943 (0.2%) in 2019 and 701 (0.2%) in 2021; *p* < 0.001). The total number of elderly patients ≥ 65 years was 513,124 (57.8%; 266,428 (56.8) in 2019 and 246,550 (59.4%) in 2021). Table 1 shows other baseline characteristics of patients transported to hospital by ambulance in Osaka Prefecture.

**Figure 1:**
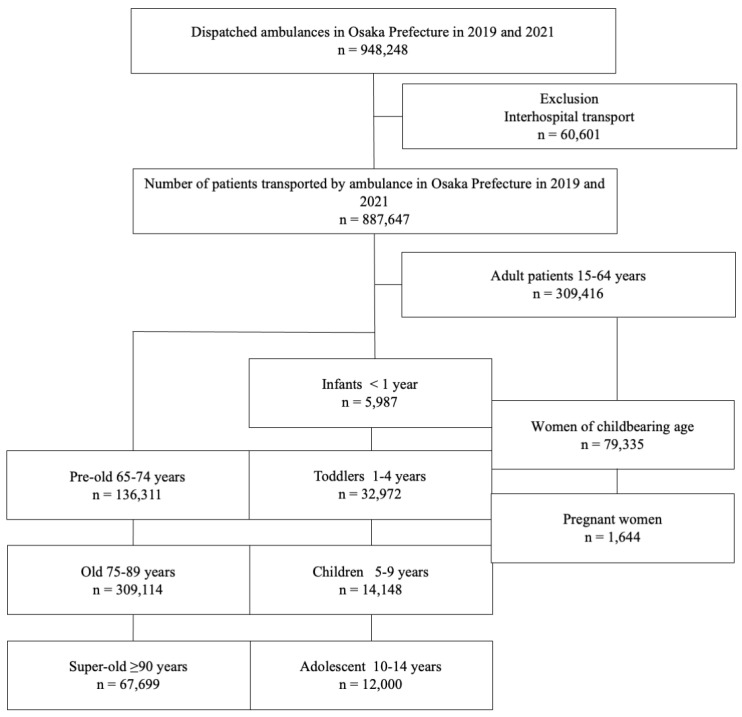
Patient flow in this study. All pediatric patients <15 years of age were divided into four groups by age. All female patients 15–44 years old were divided into those of childbearing age and those who were pregnant. All elderly patients ≥ 65 years of age were divided into three groups by age.

**Figure 2:**
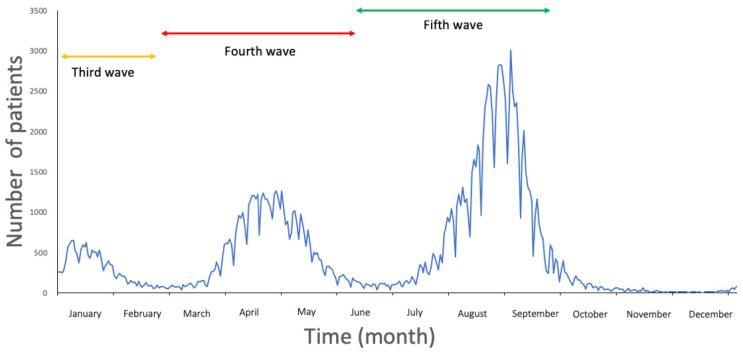
Newly diagnosed COVID-19 cases in Osaka Prefecture, Japan, in 2021. Three waves (third, fourth, and fifth waves) of the COVID-19 pandemic occurred in 2021.

Figure 1 displays the flow diagram for this study. Figure 2 shows newly diagnosed COVID-19 cases in Osaka Prefecture, which as of the time of writing had experienced five waves of infection during the COVID-19 pandemic (the first and second waves in 2020 were omitted).

Table 2 shows difficult-to-transport cases categorized according to vulnerable patients, pediatric patients, women of childbearing age, pregnant women, and elderly patients, comparing 2019 with 2021. All proportions in each category showed an increase in difficult-to-transport cases in 2021 compared with 2019. All numbers of difficult-to-transport cases except in children (5–9 y) were increased in 2021 compared with 2019.

Table 3 shows disposition at the ED among vulnerable patients, pediatric patients, women of childbearing age, pregnant women, and elderly patients. All proportions for admission to the hospital following initial arrival at the ED except those for children (5–9 y) and the super-old (≥90 y) were increased in 2021 compared with 2019. Both the number and proportion of deaths in the ED among women of childbearing age and elderly patients were increased in 2021 compared with 2019.

Table 4 shows univariate logistic regression analysis of difficult-to-transport cases among pediatric patients, women of childbearing age, pregnant women, elderly patients, and vulnerable patients, with 2019 as the reference. The results showed that difficult-to-transport cases were significantly more frequent in 2021 than in 2019, except for children (5–9 y). Difficult-to-transport cases in 2021 among non-vulnerable patients were also significantly higher than in 2019 (adjusted OR 1.28, 95%CI 1.26–1.30; *p* < 0.001), but the adjusted OR was higher for vulnerable patients than for non-vulnerable patients (adjusted OR 1.46, 95%CI 1.44–1.48; *p* < 0.001).

Table 5 shows multivariate logistic regression analysis of difficult-to-transport cases among all patients (pediatric patients, pregnant women, elderly patients, and vulnerable population as variables). Year, month, sex, weekends (compared with weekdays), and nighttime (17:00–09:00) (compared with daytime) were used as covariates. Adjusted ORs for difficult-to-transport cases among children (adjusted OR 0.33, 95%CI 0.31–0.35) and pregnant women (adjusted OR 0.48, 95%CI 0.34–0.69) were negative. The adjusted OR for difficult-to-transport cases of elderly patients was not significant (adjusted OR 1.01, 95%CI 0.99–1.03; *p* = 0.403), but elderly status represented a factor independently associated with increased risk of categorization as a difficult-to-transport case (adjusted OR 1.11, 95%CI 1.08–1.14, *p* < 0.001) in 2021 (Table S2). Difficult-to-transport cases showed a significantly lower frequency in the vulnerable population than in the non-vulnerable population (adjusted OR 0.81, 95%CI 0.80–0.83; *p* < 0.001). Supplemental tables show other results relevant to the main results. Being an adult patient (>15 and <65 years of age) was independently associated with increased risk of categorization as a difficult-to-transport case (adjusted OR 1.22, 95%CI 1.20–1.25; *p* < 0.001), regardless of study period (Table S1). COVID-19 (including suspected cases) was independently associated with increased risk of categorization as difficult-to-transport cases in all patients, regardless of variables. The highest adjusted OR for difficult-to-transport cases among vulnerable patients was nighttime (17:00–09:00). July and August were not associated with difficult-to-transport cases in 2019 but were significantly associated with difficult-to-transport cases in 2021 (Table S2).

The adjusted OR for mortality in the ED among pediatric patients was negative (adjusted OR 0.08, 95%CI 0.06–0.10; *p* < 0.001) (Table S3). The adjusted OR for mortality in the ED was positive for elderly patients (adjusted OR 4.04, 95%CI 3.84–4.26; *p* < 0.001). We analyzed adjusted ORs for mortality in the ED among elderly patients separately for 2019 and 2021 (Table 6). The adjusted OR for mortality in the ED among elderly patients was also positive in 2019 (adjusted OR 4.03, 95%CI 3.74–4.35; *p* < 0.001) and 2021 (adjusted OR 4.05, 95%CI 3.77–4.35; *p* < 0.001).

## 4. Discussion

This retrospective descriptive study found that difficulty in obtaining hospital acceptance for transfer of a vulnerable patient (difficult-to-transport cases) was increased in 2021 compared with 2019, at least in Osaka Prefecture. Status as a pediatric patients or pregnant woman were independent factors associated with decreased risk of difficult-to-transport cases, but elderly status of age ≥65 years was not independently associated with any decrease or increase in difficult-to-transport cases in the two year-long periods studied (2019 and 2021). However, the elderly population was independently associated with increased risk of difficult-to-transport cases during the COVID-19 pandemic in 2021.

The number of patients transported to hospital by ambulance was significantly lower in 2021 in Osaka than in 2019, although the number of patients transported by ambulance in Japan has been increasing for the past 10 years [18]. Similar reductions in ambulance callouts were seen all over the world during the pandemic in 2020 [19,20,21,22]. Two reasons may have contributed to this: first, transmission of COVID-19 is thought to occur through aerosolized droplets or direct contact, so some patients might have been reluctant to seek medical attention due to concerns about contracting COVID-19 after visiting a hospital. Second, less-urgent patients did not want to burden the healthcare system because of informational campaigns emphasizing the pressures on healthcare providers.

A similar analysis of 2020 found that June showed the lowest number of difficult-to-transport cases that year, and cases between July and November were not significantly associated with difficult-to-transport cases in 2019 (Table S2) [3,4]. The present study showed more difficult-to-transport cases in August and September than in June 2021, which was attributed to the fifth wave of COVID-19 in Japan (Table S2; Figure 2).

The ambulance service performs triage at the scene, selects an appropriate hospital depending on the condition of the patient, and contacts the hospital to request acceptance of the patient [23] The hospital can choose not to accept the request based on treatment capabilities and bed vacancies. High burdens on emergency care resources would obviously increase the risk of a request being declined. Before the COVID-19 pandemic, the ageing of the population has made elderly patients frequent users of ambulance services, because the EMS system including the ambulance service is fully operated by the public sector under the Japanese system of universal coverage [23]. We considered the elderly population as the main age group for difficult-to-transport cases, but this group did not show increased difficulty obtaining hospital acceptance for transport compared to before the COVID-19 pandemic (Table 5 and Table S2).

One possible reason might be that this age group had primary care physicians at hospitals near their homes, representing so-called ‘hospital-centered medical care’, and thus might have been accepted within 30 min at the nearest hospitals [24].

On the other hand, patients <65 years of age were considered more likely to be healthier and might not need the services of their primary care physician in their resident area. In addition, hospitals tended not to accept patients transported by ambulance because COVID-19 spreads more in young adults than elderly patients [25]. Such patients thus might have been unable to achieve rapid acceptance by a hospital (Table S2).

Children and pregnant women were found to have a negative association with categorization as difficult-to-transport cases, but univariate logistic analysis showed greater difficulty with hospitals accepting those patients in 2021 compared to 2019, differing from previous studies [3,4]. Each COVID-19 wave in Osaka triggered a medical crisis, where non-COVID-19 medical services such as emergency medicine were temporarily halted, so populations of children and pregnant women might have encountered difficulty with transportation [26]. There were three waves in 2021 in Japan (Figure 2). In the third COVID-19 wave, there were more infections than those in the first and second COVID-19 waves in 2020 [27]. In the fourth COVID-19 wave, the Alpha variant became the main strain of the virus [27]. In the fifth COVID-19 wave, the Delta variant became the main strain, causing a medical crisis [27]. July and August were not associated with difficult-to-transport cases in 2019 but were significantly associated with difficult-to-transport cases in 2021 (Table S2). This was due to the fifth COVID-19 wave, the Delta variant, which was associated with increased susceptibility to severe disease and resulted in increasing difficult-to-transport cases.

Elderly patients (>65 years of age) were found to have an independent association with increased mortality in the ED. The adjusted OR for death in the ED for elderly patients in 2019 was similar to that in 2021. The COVID-19 pandemic caused a medical crisis in Osaka, and old age was an independent factor associated with increased risk of difficult-to-transport cases during the COVID-19 pandemic in 2021, but this did not influence the risk of mortality in the ED. One possible reason might be that patients with serious disease using ambulance services were accepted by hospitals at similar rates in both 2019 and 2021, even during medical crises. The EMS system effectively managed serious patients in Osaka during the COVID-19 pandemic.

There was a potentially altered clinical phenotype associated with some degree of preexisting immunity conferred by COVID-19 vaccination; however, children were not considered because children’s COVID-19 vaccination started in 2022 in Japan.

Several limitations to this study need to be kept in mind. First, this study was a retrospective, observational study and thus unknown confounding factors may have impacted the results. However, the ORION system has been collecting data on all emergency patients transported to critical care centers and emergency medical institutions by ambulance in Osaka Prefecture; thus, patient selection bias is small. Second, we could not include other vulnerable populations such as those with mental illness or the homeless because ORION data do not include specific patient data due to privacy concerns. Third, the final diagnosis and prognosis in hospital were unknown since this study only analyzed ambulance records.

In the future, we will use both ambulance records and hospital data to analyze the impact of the COVID-19 pandemic on the EMS system for vulnerable patients. Our study findings will be useful for the next COVID-19 or non-COVID-19 pandemic as they provide fundamental learning regarding the epidemiology of vulnerable patients in Japan.

## 5. Conclusions

During the COVID-19 pandemic in 2021, the frequency of difficult-to-transport cases was increased compared to 2019, but vulnerable patients were not the cause of difficulty in obtaining hospital acceptance for transfer. Each wave of COVID-19 infection influenced difficult-to-transport cases. Elderly patients ≥65 years of age had an independent association with increased mortality in the ED, but not with the COVID-19 pandemic. Our study findings will be useful for other non-COVID-19 pandemics as they provide fundamental learning regarding the epidemiology of vulnerable patients.

## Figures and Tables

**Table 1:** Demographic characteristics of transported patients.

Year	2019	2021	Total	*p*-Value
Number of patients	468,697	418,950	887,647	<0.001
Age, median (IQR)	70.0 (38)	72.0 (37)		<0.001
Sex (male), %	236,661 (50.5)	212,458 (50.7)	449,119 (50.6)	0.04
Pediatric patients, %	37,547 (8.0)	27,560 (6.6)	65,107 (7.3)	<0.001
Age category of children				<0.001
Infants (0 y), %	3375 (9.0)	2612 (9.5)	5987 (9.2)	
Toddlers (1–4 y), %	18,891 (50.3)	14,081 (51.1)	32,972 (50.6)	
Children (5–9 y), %	8480 (22.6)	5668 (20.6)	14,148 (21.7)	
Adolescents (10–14 y), %	6801 (18.1)	5199 (18.9)	12,000 (18.4)	
Adult patients (15–64 y), %	164,722 (35.1)	144,694 (34.5)	309,416 (34.9)	<0.001
Women of childbearing age (15–44 y), %	43,105 (9.2)	36,230 (8.6)	79,335 (8.9)	<0.001
Pregnant women, %	943 (0.2)	701 (0.2)	1644 (0.2)	<0.001
Elderly patients, %	266,428 (56.8)	246,550 (59.4)	513,124 (57.8)	<0.001
Age category of elderly				<0.001
Pre-old (65–74 y), %	73,062 (27.4)	63,249 (25.6)	136,311 (26.6)	
Old (75–89 y), %	160,666 (60.3)	148,448 (60.2)	309,114 (60.2)	
Super-old (≥90 y), %	32,700 (12.3)	34,999 (14.2)	67,699 (13.2)	

Pediatric patients, 0–14 years; adult patients, 15–64 years; elderly patients, >65 years. Statistical analyses were performed using the χ^2^ test for categorical variables and the Kruskal–Wallis test for continuous variables. Abbreviation: IQR, interquartile range.

**Table 2:** Difficult-to-transport cases among pediatric patients, women of childbearing age, pregnant women, and elderly patients in 2019 and 2021.

2019	Not Difficult-to-Transport Cases	Difficult-to-Transport Cases	All
Infants (0 y), %	3349 (99.2)	26 (0.8)	3375 (9.0)
Toddlers (1–4 y), %	18,751 (99.3)	140 (0.7)	18,891 (50.3)
Children (5–9 y), %	8338 (98.3)	142 (1.7)	8,480 (22.6)
Adolescents (10–14 y), %	6676 (98.2)	125 (1.8)	6801 (18.1)
Total	37,114 (98.9)	433 (1.2)	37,547
2021	Not difficult-to-transport cases	Difficult-to-transport cases	All
Infants (0 y), %	2572 (98.5)	40 (1.5)	2612 (9.5)
Toddlers (1–4 y), %	13,901 (98.7)	180 (1.3)	14,081 (51.1)
Children (5–9 y), %	5548 (97.9)	120 (2.1)	5668 (20.6)
Adolescents (10–14 y), %	5071 (97.5)	128 (2.5)	5199 (18.9)
Total	27,092 (98.3)	468 (1.7)	27,560
Women of childbearing age (15–44 y), %	41,602 (96.5)	1503 (3.5)	43,105 (9.2)
Pregnant women, %	931 (98.7)	12 (1.3)	943 (0.2)
2021	Not difficult-to-transport cases	Difficult-to-transport cases	All
Women of childbearing age (15–44 y), %	34,123 (94.2)	2107 (5.8)	36,230 (8.6)
Pregnant women, %	681 (97.2)	20 (2.9)	701 (0.2)
Pre-old (65–74 y), %	71,158 (97.4)	1904 (2.6)	73,062 (27.4)
Old (75–89 y), %	156,936 (97.7)	3730 (2.3)	160,666 (60.3)
Super-old (≥90 y), %	31,806 (97.3)	894 (2.7)	32,700 (12.3)
Total	259,900 (97.6)	6528 (2.5)	266,428
2021	Not difficult-to-transport cases	Difficult-to-transport cases	All
Pre-old (65–74 y), %	60,320 (95.4)	2929 (4.6)	63,249 (25.6)
Old (75–89 y), %	140,982 (95.0)	7466 (5.0)	148,448 (60.2)
Super-old (≥90 y), %	32,838 (93.8)	2161 (6.2)	34,999 (14.2)
Total	234,140 (94.9)	12,556 (5.1)	246,696

**Table 3:** Disposition at the emergency department among pediatric patients, women of childbearing age, pregnant women, and elderly patients.

Pediatric Patients					
2019	Infants (0 y), %	Toddlers (1–4 y), %	Children (5–9 y), %	Adolescents (10–14 y), %	Total, %
Admission, %	650 (19.3)	3103 (16.4)	1410 (16.6)	1023 (15.0)	6186 (16.5)
Home, %	2682 (79.5)	15,719 (83.2)	7020 (82.8)	5722 (84.1)	31,143 (82.9)
Transfer, %	21 (0.6)	55 (0.3)	48 (0.6)	48 (0.7)	172 (0.5)
Death, %	21 (0.6)	14 (0.1)	2 (0)	8 (0.1)	45 (0.1)
No show, %	1 (0)	0	0	0	1 (0)
Total	3375	18,891	8480	6801	37,547
2021	Infants (0 y), %	Toddlers (1–4 y), %	Children (5–9 y), %	Adolescents (10–14 y), %	Total, %
Admission, %	498 (19.3)	2429 (17.3)	929 (16.4)	908 (17.5)	4764 (17.3)
Home, %	2074 (79.4)	11,595 (82.4)	4702 (83.0)	4245 (81.7)	22,616 (82.1)
Transfer, %	27 (1.0)	53 (0.4)	36 (0.6)	41 (0.8)	157 (0.6)
Death, %	12 (0.5)	4 (0)	1 (0)	5 (0)	22 (0.1)
No show, %	1 (0)	0	0	0	1 (0)
Total	2612	14,081	5668	5199	27,560
Women of childbearing age and pregnant women					
2019	Pregnant women, %		Women of childbearing age (15–44 y), %		
Admission, %	547 (58.0)		6938 (16.1)		
Home, %	391 (41.5)		35,807 (83.1)		
Transfer, %	5 (0.5)		283 (0.7)		
Death, %	0		66 (0.2)		
No show, %	0		11 (0.0)		
Total	943		43,105		
2021	Pregnant women, %		Women of childbearing age (15–44 y), %		
Admission, %	418 (59.6)		6086 (16.8)		
Home, %	274 (39.1)		29,777 (82.2)		
Transfer, %	9 (1.3)		250 (0.7)		
Death, %	0		111 (0.3)		
No show, %	0		6 (0.0)		
Total	943		36,230		
Elderly patients					
2019	Pre-old (65–74 y), %		Old (75–89 y), %	Super-old (≥90 y), %	Total, %
Admission, %	31,161 (42.7)		80,713 (50.2)	20,128 (61.6)	132,002 (49.6)
Home, %	39,904 (54.6)		74,514 (46.4)	11,162 (34.1)	125,580 (47.1)
Transfer, %	1163 (1.6)		2991 (1.9)	593 (1.8)	4747 (1.8)
Death, %	827 (1.1)		2443 (1.5)	816 (2.5)	4086 (1.5)
No show, %	7 (0)		5 (0)	1 (0)	13 (0)
Total	73,062		160,666	32,700	266,428
2021	Pre-old (65–74 y), %		Old (75–89 y), %	Super-old (≥90 y), %	Total, %
Admission, %	29,369 (46.4)		77,541 (52.2)	21,529 (61.5)	128,439 (52.1)
Home, %	31,761 (50.2)		64,981 (43.8)	11,677 (33.4)	108,419 (44.0)
Transfer, %	1163 (1.8)		2991 (2.0)	733 (2.1)	4887 (2.0)
Death, %	952 (1.5)		2927 (2.0)	1057 (3.0)	4936 (2.0)
No show, %	4 (0)		8 (0)	3 (0)	15 (0)
Total	63,249		148,448	34,999	246,696

**Table 4:** Univariate logistic regression analysis of difficult-to-transport cases among pediatric patients, women of childbearing age, pregnant women, elderly patients, and vulnerable patients as a variable.

2021 vs. 2019 (Reference)					
	Odds Ratio	95%CI			*p*-Value
Infants (0 y)	1.42	1.10	–	1.81	0.006
Toddlers (1–4 y)	1.32	1.18	–	1.47	<0.001
Children (5–9 y)	1.13	1.00	–	1.27	0.056
Adolescents (10–14 y)	1.16	1.03	–	1.32	0.019
Women of childbearing age (15–44 y)	1.31	1.26	–	1.35	<0.001
Pregnant women	1.51	1.05	–	2.17	0.025
Pre-old (65–74 y)	1.35	1.31	–	1.39	<0.001
Old (75–89 y)	1.49	1.46	–	1.52	<0.001
Super-old (≥90 y)	1.53	1.47	–	1.59	<0.001
Non-vulnerable patients	1.28	1.26	–	1.30	<0.001
Vulnerable patients	1.46	1.44	–	1.48	<0.001

**Table 5:** Multivariate logistic regression analysis of difficult-to-transport cases among all patients (pediatric patients, pregnant women, elderly patients, and vulnerable population as a variable).

Pediatric Patients as a Variable					
	Odds Ratio	95%CI			*p*-Value
Year					
2019	Reference				
2021	1.39	1.37	–	1.40	<0.001
Female (compared with male)	0.91	0.89	–	0.93	<0.001
Month					
June	Reference				
January	1.87	1.78	–	1.98	<0.001
February	1.52	1.44	–	1.61	<0.001
March	1.24	1.17	–	1.32	<0.001
April	1.64	1.56	–	1.74	<0.001
May	1.59	1.50	–	1.68	<0.001
July	1.00	0.94	–	1.06	0.963
August	1.28	1.21	–	1.35	<0.001
September	1.27	1.20	–	1.35	<0.001
October	1.01	0.95	–	1.07	0.851
November	1.01	0.95	–	1.07	0.773
December	1.06	1.00	–	1.13	0.04
Weekends (compared with weekdays)	1.19	1.16	–	1.22	<0.001
Nighttime (17:00–09:00) (compared with daytime)	2.55	2.49	–	2.61	<0.001
Children (compared with adult > 15 y)	0.33	0.31	–	0.35	<0.001
COVID-19 (including suspected cases)	1.38	1.29	–	1.46	<0.001
Pregnant women as a variable					
	Odds ratio	95%CI			*p*-value
Year					
2019	Reference				
2021	1.39	1.38	–	1.41	<0.001
Female (compared with male)	0.93	0.91	–	0.95	<0.001
Month					
June	Reference				
January	1.91	1.81	–	2.01	<0.001
February	1.55	1.47	–	1.65	<0.001
March	1.27	1.20	–	1.34	<0.001
April	1.66	1.57	–	1.75	<0.001
May	1.60	1.51	–	1.69	<0.001
July	1.01	0.95	–	1.07	0.752
August	1.31	1.23	–	1.38	<0.001
September	1.29	1.22	–	1.37	<0.001
October	1.02	0.96	–	1.09	0.448
November	1.03	0.97	–	1.09	0.365
December	1.08	1.02	–	1.15	0.008
Weekends (compared with weekdays)	1.18	1.16	–	1.21	<0.001
Nighttime (17:00–09:00) (compared with daytime)	2.53	2.47	–	2.59	<0.001
Pregnant women	0.48	0.34	–	0.69	<0.001
COVID-19 (including suspected cases)	1.44	1.35	–	1.53	<0.001
Elderly patients as a variable					
	Odds ratio	95%CI			*p*-value
Year					
2019	Reference				
2021	1.39	1.38	–	1.41	<0.001
Female (compared with male)	0.93	0.91	–	0.95	<0.001
Month					
June	Reference				
January	1.91	1.81	–	2.01	<0.001
February	1.55	1.47	–	1.65	<0.001
March	1.27	1.20	–	1.34	<0.001
April	1.66	1.57	–	1.75	<0.001
May	1.60	1.51	–	1.69	<0.001
July	1.01	0.95	–	1.07	0.755
August	1.31	1.23	–	1.38	<0.001
September	1.29	1.22	–	1.37	<0.001
October	1.02	0.96	–	1.09	0.453
November	1.03	0.97	–	1.09	0.373
December	1.08	1.02	–	1.15	0.008
Weekends (compared with weekdays)	1.18	1.16	–	1.21	<0.001
Nighttime (17:00–09:00) (compared with daytime)	2.53	2.47	–	2.59	<0.001
Old (compared with young <65 y)	1.01	0.99	–	1.03	0.403
COVID-19 (including suspected cases)	1.44	1.35	–	1.53	<0.001
Vulnerable patients as a variable					
	Odds ratio	95%CI			*p*-value
Year					
2019	Reference				
2021	1.40	1.38	–	1.41	<0.001
Female (compared with male)	0.94	0.92	–	0.96	<0.001
Month					
June	Reference				
January	1.93	1.83	–	2.03	<0.001
February	1.56	1.48	–	1.65	<0.001
March	1.27	1.20	–	1.35	<0.001
April	1.66	1.57	–	1.75	<0.001
May	1.60	1.51	–	1.69	<0.001
July	1.01	0.95	–	1.07	0.867
August	1.29	1.22	–	1.37	<0.001
September	1.29	1.22	–	1.37	<0.001
October	1.02	0.96	–	1.09	0.457
November	1.03	0.97	–	1.10	0.328
December	1.09	1.02	–	1.15	0.006
Weekends (compared with weekdays)	1.18	1.16	–	1.21	<0.001
Nighttime (17:00–09:00) (compared with daytime)	2.47	2.41	–	2.53	<0.001
Vulnerable patients (compared with non-vulnerable)	0.81	0.80	–	0.83	<0.001
COVID-19 (including suspected cases)	1.41	1.32	–	1.50	<0.001

**Table 6:** Multivariate logistic regression analysis of death in the ED for all patients in 2019 and 2021 (elderly patients as a variable).

	**Odds Ratio**	**95%CI**			***p*** **-Value**
Year					
2019	Reference				
2021	1.17	1.15	–	1.19	<0.001
Female (compared with male)	0.76	0.73	–	0.79	<0.001
Month					
June	Reference				
January	1.62	1.47	–	1.78	<0.001
February	1.52	1.38	–	1.68	<0.001
March	1.24	1.12	–	1.37	<0.001
April	1.30	1.18	–	1.44	<0.001
May	1.32	1.20	–	1.46	<0.001
July	0.92	0.82	–	1.02	0.103
August	0.99	0.89	–	1.10	0.845
September	1.05	0.94	–	1.16	0.387
October	1.02	0.92	–	1.14	0.659
November	1.19	1.08	–	1.32	0.001
December	1.37	1.24	–	1.50	<0.001
Weekends (compared with weekdays)	0.98	0.93	–	1.02	0.242
Nighttime (17:00–09:00) (compared with daytime)	1.60	1.54	–	1.66	<0.001
Old (compared with young <65 y)	4.04	3.83	–	4.25	<0.001
COVID-19 (including suspected cases)	0.25	0.19	–	0.32	<0.001
2019					
	Odds ratio	95% confidence interval			*p*-value
Female (compared with male)	0.74	0.70	–	0.78	<0.001
Month					
June	Reference				
January	1.54	1.35	–	1.76	<0.001
February	1.51	1.31	–	1.73	<0.001
March	1.27	1.10	–	1.47	0.001
April	1.17	1.01	–	1.35	0.039
May	1.06	0.91	–	1.23	0.446
July	0.88	0.76	–	1.03	0.115
August	0.85	0.73	–	0.99	0.041
September	1.02	0.87	–	1.18	0.821
October	0.99	0.85	–	1.16	0.938
November	1.17	1.01	–	1.35	0.038
December	1.32	1.15	–	1.52	<0.001
Weekends (compared with weekdays)	0.94	0.88	–	1.00	0.046
Nighttime (17:00–09:00) (compared with daytime)	1.54	1.46	–	1.64	<0.001
Old (compared with young <65 y)	4.03	3.74	–	4.34	<0.001
COVID-19 (including suspected cases)	(omitted)				
2021					
	Odds ratio	95% confidence interval			*p*-value
Female (compared with male)	0.78	0.74	–	0.83	<0.001
Month					
June	Reference				
January	1.68	1.48	–	1.91	<0.001
February	1.54	1.34	–	1.76	<0.001
March	1.21	1.06	–	1.39	0.006
April	1.42	1.24	–	1.63	<0.001
May	1.59	1.38	–	1.82	<0.001
July	0.94	0.82	–	1.09	0.432
August	1.12	0.97	–	1.29	0.11
September	1.07	0.93	–	1.24	0.345
October	1.05	0.91	–	1.21	0.512
November	1.22	1.06	–	1.40	0.005
December	1.41	1.24	–	1.61	<0.001
Weekends (compared with weekdays)	1.00	0.95	–	1.06	0.974
Nighttime (17:00–09:00) (compared with daytime)	1.64	1.56	–	1.73	<0.001
Old (compared with young <65 y)	4.05	3.77	–	4.34	<0.001
COVID-19 (including suspected cases)	0.24	0.19		0.32	<0.001

## Data Availability

The datasets used and/or analyzed during the current study are available from the corresponding author on reasonable request.

## Supplementary Materials

The following supporting information can be downloaded at https://www.mdpi.com/article/10.3390/medicina60020345/s1. Table S1: Multivariate logistic regression analysis of difficult-to-transport cases among all patients (adult as a variable); Table S2: Multivariate logistic regression analysis of difficult-to-transport cases among all patients in 2019 and 2021 (pediatric patients, pregnant women, elderly patients, and vulnerable population as a variable); Table S3: Multivariate logistic regression analysis of death in the ED for all patients (pediatric patients as a variable).
