# Supplementary material for: Influence of the COVID-19 Outbreak in Vulnerable Patients (Pediatric Patients, Pregnant Women, and Elderly Patients) on an Emergency Medical Service System: A Pre- and Post-COVID-19 Pandemic Comparative Study Using the Population-Based ORION Registry
Source: Medicina (Kaunas). 2024 Feb 19;60(2):345. doi: 10.3390/medicina60020345 (PMC10890565; doi:10.3390/medicina60020345)
Supplement: Supplementary file 1 [file medicina-60-00345-s001.zip › medicina-2857784-supplementary.pdf]

medicina

MDPI

*Article*

# Influence of the COVID-19 Outbreak in Vulnerable Patients (Pediatric Patients, Pregnant Women, and Elderly Patients) on an Emergency Medical Service System: A Pre- and Post-COVID-19 Pandemic Comparative Study Using the Population-Based ORION Registry

**Koshi Ota [1,2,*], Masahiko Nitta [1], Tomonobu Komeya [3], Tetsuya Matsuoka [2] and Akira Takasu [1]**

[1] Department of Emergency and Critical Care Medicine, Osaka Medical and Pharmaceutical University, Takatsuki 569-8686, Japan; nittam@ompu.ac.jp (M.N.); akira.takasu@ompu.ac.jp (A.T.)
[2] Working Group for Analysis of the Emergency Medical Care System in Osaka Prefecture, Osaka 530-0000, Japan; t-matsuoka@rgmc.izumisano.osaka.jp
[3] Osaka Prefectural Government, Osaka 540-8570, Japan; komeyat@mbox.pref.osaka.lg.jp
* Correspondence: koshi.ota@ompu.ac.jp

**Table S1.** Multivariate logistic regression analysis of difficult-to-transport cases among all patients (adult as a variable).

| | **Odds Ratio** | **95%CI** | | | ***p*-Value** |
|---|---|---|---|---|---|
| Year | | | | | |
| 2019 | Reference | | | | |
| 2021 | 1.40 | 1.38 | – | 1.41 | <0.001 |
| Female (compared with male) | 0.94 | 0.92 | – | 0.96 | <0.001 |
| Month | | | | | |
| June | Reference | | | | |
| January | 1.93 | 1.83 | – | 2.03 | <0.001 |
| February | 1.56 | 1.48 | – | 1.65 | <0.001 |
| March | 1.27 | 1.20 | – | 1.35 | <0.001 |
| April | 1.66 | 1.57 | | 1.75 | <0.001 |
| May | 1.60 | 1.51 | | 1.69 | <0.001 |
| July | 1.01 | 0.95 | | 1.07 | 0.866 |
| August | 1.29 | 1.22 | | 1.37 | <0.001 |
| September | 1.29 | 1.22 | | 1.37 | <0.001 |
| October | 1.02 | 0.96 | | 1.09 | 0.457 |
| November | 1.03 | 0.97 | | 1.10 | 0.329 |
| December | 1.09 | 1.02 | | 1.15 | 0.006 |
| Weekends (compared with weekdays) | 1.18 | 1.16 | – | 1.21 | <0.001 |
| Nighttime (17:00–09:00) (compared with daytime) | 2.47 | 2.41 | – | 2.53 | <0.001 |
| Adult (age >15 y and <65 y) | 1.22 | 1.20 | – | 1.25 | <0.001 |
| COVID-19 (including suspected cases) | 1.41 | 1.33 | – | 1.50 | <0.001 |

**Table S2.** Multivariate logistic regression analysis of difficult-to-transport cases among all patients in 2019 and 2021 (pediatric patients, pregnant women, elderly patients, and vulnerable population as a variable).

| **Pediatric patients as a variable** | | | |
|---|---|---|---|
| **2019** | | | |
| | **Odds ratio** | **95%CI** | ***P*-value** |

| | | | | | |
|---|---|---|---|---|---|
| Female (compared with male) | 0.88 | 0.85 | – | 0.91 | <0.001 |
| Month | | | | | |
| June | Reference | | | | |
| January | 2.03 | 1.87 | – | 2.20 | <0.001 |
| February | 1.65 | 1.51 | – | 1.80 | <0.001 |
| March | 1.38 | 1.26 | – | 1.51 | <0.001 |
| April | 1.26 | 1.15 | – | 1.38 | <0.001 |
| May | 1.17 | 1.06 | – | 1.28 | 0.001 |
| July | 0.95 | 0.86 | – | 1.04 | 0.25 |
| August | 1.06 | 0.97 | – | 1.16 | 0.196 |
| September | 0.94 | 0.85 | – | 1.04 | 0.219 |
| October | 0.98 | 0.89 | – | 1.08 | 0.629 |
| November | 1.02 | 0.93 | – | 1.12 | 0.711 |
| December | 1.11 | 1.01 | – | 1.21 | 0.03 |
| Weekends (compared with weekdays) | 1.24 | 1.19 | – | 1.29 | <0.001 |
| Nighttime (17:00–09:00) (compared with daytime) | 2.95 | 2.83 | – | 3.08 | <0.001 |
| Children (compared with adult >15 y) | 0.37 | 0.34 | – | 0.41 | <0.001 |
| COVID-19 (including suspected cases) | (omitted) | | | | |
| | | | | | |
| 2021 | | | | | |
| Female (compared with male) | 0.93 | 0.90 | – | 0.96 | <0.001 |
| Month | | | | | |
| June | Reference | | | | |
| January | 1.75 | 1.63 | – | 1.88 | <0.001 |
| February | 1.44 | 1.33 | – | 1.55 | <0.001 |
| March | 1.16 | 1.08 | – | 1.25 | <0.001 |
| April | 1.92 | 1.79 | – | 2.07 | <0.001 |
| May | 1.91 | 1.78 | – | 2.06 | <0.001 |
| July | 1.04 | 0.96 | – | 1.12 | 0.319 |
| August | 1.44 | 1.34 | – | 1.55 | <0.001 |
| September | 1.50 | 1.40 | – | 1.62 | <0.001 |
| October | 1.03 | 0.96 | – | 1.12 | 0.411 |
| November | 1.01 | 0.93 | – | 1.09 | 0.873 |
| December | 1.04 | 0.96 | – | 1.12 | 0.332 |
| Weekends (compared with weekdays) | 1.16 | 1.12 | – | 1.19 | <0.001 |
| Nighttime (17:00–09:00) (compared with daytime) | 2.35 | 2.28 | – | 2.42 | <0.001 |
| Pediatric patients (compared with adult >15 y) | 0.29 | 0.27 | – | 0.32 | <0.001 |
| COVID-19 (including suspected cases) | 1.30 | 1.22 | – | 1.38 | <0.001 |
| | | | | | |
| Pregnant women as a variable | | | | | |
| 2019 | | | | | |
| Female (compared with male) | 0.90 | 0.87 | – | 0.93 | <0.001 |
| Month | | | | | |
| June | Reference | | | | |
| January | 2.03 | 1.87 | – | 2.20 | <0.001 |
| February | 1.66 | 1.52 | – | 1.82 | <0.001 |
| March | 1.39 | 1.27 | – | 1.52 | <0.001 |
| April | 1.27 | 1.16 | – | 1.39 | <0.001 |
| May | 1.17 | 1.07 | – | 1.28 | 0.001 |
| July | 0.95 | 0.86 | – | 1.05 | 0.306 |
| August | 1.08 | 0.99 | – | 1.18 | 0.1 |

| | | | | | |
|---|---|---|---|---|---|
| September | 0.95 | 0.87 | – | 1.05 | 0.34 |
| October | 0.99 | 0.90 | – | 1.09 | 0.863 |
| November | 1.04 | 0.94 | – | 1.14 | 0.47 |
| December | 1.12 | 1.02 | – | 1.22 | 0.018 |
| Weekends (compared with weekdays) | 1.23 | 1.19 | – | 1.28 | <0.001 |
| Nighttime (17:00–09:00) (compared with daytime) | 2.93 | 2.82 | – | 3.06 | <0.001 |
| Pregnant women | 0.44 | 0.25 | – | 0.77 | 0.004 |
| COVID-19 (including suspected cases) | (omitted) | | | | |
| | | | | | |
| 2021 | | | | | |
| Female (compared with male) | 0.95 | 0.92 | – | 0.98 | <0.001 |
| Month | | | | | |
| June | Reference | | | | |
| January | 1.81 | 1.68 | – | 1.94 | <0.001 |
| February | 1.48 | 1.37 | – | 1.59 | <0.001 |
| March | 1.19 | 1.10 | – | 1.28 | <0.001 |
| April | 1.94 | 1.80 | – | 2.08 | <0.001 |
| May | 1.92 | 1.79 | – | 2.07 | <0.001 |
| July | 1.05 | 0.98 | – | 1.14 | 0.172 |
| August | 1.47 | 1.37 | – | 1.58 | <0.001 |
| September | 1.54 | 1.43 | – | 1.66 | <0.001 |
| October | 1.05 | 0.97 | – | 1.14 | 0.189 |
| November | 1.03 | 0.95 | – | 1.11 | 0.498 |
| December | 1.06 | 0.98 | – | 1.15 | 0.116 |
| Weekends (compared with weekdays) | 1.15 | 1.12 | – | 1.18 | <0.001 |
| Nighttime (17:00–09:00) (compared with daytime) | 2.32 | 2.26 | – | 2.40 | <0.001 |
| Pregnant women | 0.52 | 0.33 | – | 0.81 | 0.004 |
| COVID-19 (including suspected cases) | 1.36 | 1.28 | – | 1.45 | <0.001 |
| | | | | | |
| Elderly patients as a variable | | | | | |
| 2019 | | | | | |
| Female (compared with male) | 0.91 | 0.87 | – | 0.94 | <0.001 |
| Month | | | | | |
| June | Reference | | | | |
| January | 2.04 | 1.88 | – | 2.22 | <0.001 |
| February | 1.67 | 1.53 | – | 1.83 | <0.001 |
| March | 1.40 | 1.28 | – | 1.53 | <0.001 |
| April | 1.27 | 1.16 | – | 1.39 | <0.001 |
| May | 1.17 | 1.07 | – | 1.29 | 0.001 |
| July | 0.95 | 0.86 | – | 1.05 | 0.299 |
| August | 1.08 | 0.99 | – | 1.18 | 0.095 |
| September | 0.96 | 0.87 | – | 1.05 | 0.357 |
| October | 0.99 | 0.90 | – | 1.10 | 0.915 |
| November | 1.04 | 0.95 | – | 1.15 | 0.399 |
| December | 1.12 | 1.02 | – | 1.23 | 0.015 |
| Weekends (compared with weekdays) | 1.23 | 1.18 | – | 1.28 | <0.001 |
| Nighttime (17:00–09:00) (compared with daytime) | 2.89 | 2.77 | – | 3.01 | <0.001 |
| Old (compared with young <65 y) | 0.87 | 0.84 | – | 0.90 | <0.001 |
| COVID-19 (including suspected cases) | (omitted) | | | | |
| 2021 | | | | | |
| Female (compared with male) | 0.94 | 0.91 | – | 0.97 | <0.001 |

| | | | | | |
|---|---|---|---|---|---|
| Month | | | | | |
| June | Reference | | | | |
| January | 1.79 | 1.67 | – | 1.92 | <0.001 |
| February | 1.47 | 1.36 | – | 1.58 | <0.001 |
| March | 1.18 | 1.10 | – | 1.28 | <0.001 |
| April | 1.93 | 1.80 | – | 2.08 | <0.001 |
| May | 1.92 | 1.79 | – | 2.06 | <0.001 |
| July | 1.06 | 0.98 | – | 1.14 | 0.165 |
| August | 1.48 | 1.37 | – | 1.59 | <0.001 |
| September | 1.54 | 1.43 | – | 1.66 | <0.001 |
| October | 1.05 | 0.97 | – | 1.14 | 0.208 |
| November | 1.02 | 0.95 | – | 1.11 | 0.556 |
| December | 1.06 | 0.98 | – | 1.14 | 0.144 |
| Weekends (compared with weekdays) | 1.15 | 1.12 | – | 1.19 | <0.001 |
| Nighttime (17:00–09:00) (compared with daytime) | 2.35 | 2.28 | – | 2.42 | <0.001 |
| Old (compared with young <65 y) | 1.11 | 1.08 | – | 1.14 | <0.001 |
| COVID-19 (including suspected cases) | 1.37 | 1.29 | – | 1.46 | <0.001 |
| | | | | | |
| Vulnerable patients as a variable | | | | | |
| 2019 | | | | | |
| | **Odds ratio** | **95%CI** | | | ***P*-value** |
| Female (compared with male) | 0.91 | 0.88 | – | 0.95 | <0.001 |
| Month | | | | | |
| June | Reference | | | | |
| January | 2.07 | 1.90 | – | 2.24 | <0.001 |
| February | 1.68 | 1.54 | – | 1.83 | <0.001 |
| March | 1.40 | 1.28 | – | 1.53 | <0.001 |
| April | 1.27 | 1.16 | – | 1.39 | <0.001 |
| May | 1.17 | 1.07 | – | 1.29 | 0.001 |
| July | 0.95 | 0.86 | – | 1.04 | 0.266 |
| August | 1.07 | 0.98 | – | 1.18 | 0.13 |
| September | 0.95 | 0.86 | – | 1.05 | 0.308 |
| October | 0.99 | 0.90 | – | 1.09 | 0.877 |
| November | 1.04 | 0.95 | – | 1.15 | 0.384 |
| December | 1.12 | 1.02 | – | 1.23 | 0.014 |
| Weekends (compared with weekdays) | 1.23 | 1.18 | – | 1.28 | <0.001 |
| Nighttime (17:00–09:00) (compared with daytime) | 2.82 | 2.70 | – | 2.93 | <0.001 |
| Vulnerable patients (compared with non-vulnerable) | 0.69 | 0.67 | – | 0.72 | <0.001 |
| COVID-19 (including suspected cases) | (omitted) | | | | |
| 2021 | | | | | |
| Female (compared with male) | 0.95 | 0.93 | – | 0.98 | <0.001 |
| Month | | | | | |
| June | Reference | | | | |
| January | 1.81 | 1.69 | – | 1.95 | <0.001 |
| February | 1.48 | 1.37 | – | 1.60 | <0.001 |
| March | 1.19 | 1.10 | – | 1.28 | <0.001 |
| April | 1.94 | 1.81 | – | 2.08 | <0.001 |
| May | 1.92 | 1.79 | – | 2.07 | <0.001 |
| July | 1.05 | 0.97 | – | 1.14 | 0.196 |

| | | | | | |
|---|---|---|---|---|---|
| August | 1.46 | 1.36 | – | 1.57 | <0.001 |
| September | 1.54 | 1.43 | – | 1.65 | <0.001 |
| October | 1.05 | 0.97 | – | 1.14 | 0.193 |
| November | 1.03 | 0.95 | – | 1.11 | 0.489 |
| December | 1.06 | 0.99 | – | 1.15 | 0.108 |
| Weekends (compared with weekdays) | 1.15 | 1.12 | – | 1.18 | <0.001 |
| Nighttime (17:00–09:00) (compared with daytime) | 2.30 | 2.23 | – | 2.37 | <0.001 |
| Vulnerable patients (compared with non-vulnerable) | 0.90 | 0.88 | – | 0.93 | <0.001 |
| COVID-19 (including suspected cases) | 1.35 | 1.27 | – | 1.44 | <0.001 |

**Table S3.** Multivariate logistic regression analysis of death in the ED for all patients (pediatric patients as a variable).

| | **Odds ratio** | **95% confidence interval** | | | ***p*-Value** |
|---|---|---|---|---|---|
| Year | | | | | |
| 2019 | Reference | | | | |
| 2021 | 1.17 | 1.15 | – | 1.20 | <0.001 |
| Female (compared with male) | 0.80 | 0.77 | – | 0.83 | <0.001 |
| Season | | | | | |
| Spring | Reference | | | | |
| Summer | 0.73 | 0.69 | – | 0.78 | <0.001 |
| Autumn | 0.84 | 0.80 | – | 0.89 | <0.001 |
| Winter | 1.19 | 1.13 | – | 1.25 | <0.001 |
| Weekends (compared with weekdays) | 0.97 | 0.93 | – | 1.01 | 0.18 |
| Nighttime (17:00–09:00) (compared with daytime) | 1.41 | 1.35 | – | 1.46 | <0.001 |
| Children (compared with adult >15y) | 0.08 | 0.06 | – | 0.10 | <0.001 |
| Covid-19 (including suspected cases) | 0.23 | 0.18 | – | 0.30 | <0.001 |
